# Temperature Effects on Development of *Meloidogyne Enterolobii* and *M. Floridensis*

**DOI:** 10.2478/jofnem-2022-0013

**Published:** 2022-06-10

**Authors:** Jeanny A. Velloso, Mary Ann D. Maquilan, Vicente P. Campos, Janete A. Brito, Donald W. Dickson

**Affiliations:** 1 Department of Plant Pathology, Laboratory of Nematology, Universidade Federal de Lavras (UFLA), Lavras, Minas Gerais 37200-900, Brazil; 2 Entomology and Nematology Department, University of Florida, Gainesville, FL 32611 United State; 3 Institude of Plant Breeding, College of Agriculture and Food Science, University of the Philippines Los Banos, Laguna 4031, Philippines; 4 Florida Department of Agriculture and Consumer Services, Division of Plant Industry, Gainesville, FL 32614-7100 United State

**Keywords:** degree-day, development, ecology, life cycle, pacara earpod tree root-knot nematode, peach root-knot nematode, temperature

## Abstract

*Meloidogyne enterolobii* and *M. floridensis* are virulent species that can overcome root-knot nematode resistance in economically important crops. Our objectives were to determine the effects of temperature on the infectivity of second-stage juveniles (J2) of these two species and determine differences in duration and thermal-time requirements (degree-days [DD]) to complete their developmental cycle. Florida isolates of *M*. *enterolobii* and *M*. *floridensis* were compared to *M. incognita* race 3. Tomato cv. BHN 589 seedlings following inoculation were placed in growth chambers set at constant temperatures of 25°C, and 30°C, and alternating temperatures of 30°C to 25°C (day–night). Root infection by the three nematode species was higher at 30°C than at 25°C, and intermediate at 30°C to 25°C, with 33%, 15%, and 24% infection rates, respectively. There was no difference, however, in the percentages of J2 that infected roots among species at each temperature. Developmental time from infective J2 to reproductive stage for the three species was shorter at 30°C than at 25°C, and 30°C to 25°C. The shortest time and DD to egg production for the three species were 13 days after inoculation (DAI) and 285.7 DD, respectively. During the experimental timeframe of 29 d, a single generation was completed at 30°C for all three species, whereas only *M. floridensis* completed a generation at 30°C to 25°C. The number of days and accumulated DD for completing the life cycle (from J2 to J2) were 23 d and 506.9 DD for *M*. *enterolobii*, and 25 d and 552.3 DD for *M. floridensis* and *M. incognita*, respectively. Exposure to lower (25°C) and intermediate temperatures (30°C to 25°C) decreased root penetration and slowed the developmental cycle of *M. enterolobii* and *M. floridensis* compared with 30°C.

*Meloidogyne enterolobii* (= *M. mayaguensis*) is an emerging root-knot nematode (RKN) species that is highly virulent on agricultural crops (Castagnone-Sereno, 2012) including many economically important vegetable and field crops (Yang and Eisenback, 1983; Carneiro et al., 2001; Brito et al., 2014; Villar-Luna et al., 2016). This nematode species has been increasingly detected worldwide predominantly in warmer climates, with the highest reported occurrence in South America, and thus it is now considered as a major threat to crop production (Philbrick et al., 2020; Collett et al., 2021). In addition to its high reproduction rate and causing severe root galling on host roots, *M. enterolobii* is also a cause for concern due to its ability to develop on crops that are typically resistant to *M. arenaria*, *M. incognita*, and *M. javanica* (Castagnone-Sereno, 2012). Some RKN-resistant crops that are known to be affected by *M*. *enterolobii* include sweet potato, soybean (Fargette and Braaksma, 1990), tomato (*Mi*-1 gene), bell pepper (*N* gene), and sweet pepper (*Tabasco* gene) (Brito et al., 2007; Kiewnick et al., 2009). Recently, it was also found to infect RKN-resistant sweet potato in North Carolina, South Carolina, and Louisiana (Ye, et al., 2013; Anonymous 2018; Rutter et al., 2019), and soybean and cotton in North Carolina (Ye, et al., 2013). *M. enterolobii* has been reported to infect many economically important crops in Brazil such as guava (Carneiro et al., 2001), and most recently, sweet potato (Silva, et al., 2021).

The resistance-breaking ability of *M. floridensis* (peach RKN) on economically important crops (Handoo et al., 2004) suggests this species as an important pathogen in agriculture, but which has so far been reported only in the USA. *M. floridensis* is of concern in Florida agriculture because of its ability to reproduce on cvs. Nemaguard, Okinawa, Nemared, and Guardian peach rootstocks (Nyczepir et al., 1998; Stanley et al., 2009), which are resistant to both *M. javanica* and *M. incognita* (Sharpe et al., 1969; Sherman et al., 1991). A virulent isolate of *M. floridensis* (MFGnv14) was found recently infecting peach rootstock, cv. Flordaguard (Maquilan et al., 2018; Qiu et al., 2022). Flordaguard rootstock was bred specifically to ensure root-knot disease protection for the Florida peach industry (Sherman et al., 1991). Field infestations of *M. floridensis* were first noted on tomato (Church, 2007), and later cucumber, eggplant, snap bean, and lilac tasselflower (*Emilia sonchifolia*) (Brito et al., 2005). In addition to Florida, there have been reports of this nematode severely infecting RKN-resistant peach-almond hybrid rootstock, Hansen 536 and Bright’s Hybrid^®^5 in California (Westphal et al., 2019), peach rootstock cv. Guardian in South Carolina orchards (Reighard et al., 2019), and tomato in Georgia (Marquez et al., 2020).

Temperature is an important factor affecting nematode development, infection rates, reproduction, survival, and migration (Madulu and Trudgill, 1994; Trudgill and Perry, 1994; Thompson et al., 2015; Leitao et al., 2021). Studies on thermal requirements of RKNs are important because of the poikilothermic nature of these pathogens, whereby temperature has a direct influence on their ecological adaptation (Trudgill et al., 2005). Thermal requirements of RKN species have been reported for *M. incognita* (Triantaphyllou and Hirschmann, 1960), *M. hapla* (Trudgill and Perry, 1994), *M. javanica* and *M. arenaria*, (Madulu and Trudgill, 1994; Dávila-Negrón and Dickson, 2013), *M. hispanica* (Maleita et al., 2012), and *M. chitwoodi* and *M. fallax* (Khan et al., 2014). However, for both emerging species *M. enterolobii* and *M. floridensis*, the temperature required for their development and life cycle completion is unknown. A recent review reports that there has been little research reported on the biology of *M. enterolobii* (Collett et al., 2021). Therefore, the objectives of the present study were to determine the effects of temperature on the life cycle and temporal variations of progression from infective to reproductive stage and emergence of second generation of J2 of *M. enterolobii*, concurrently with *M. floridensis* and compared to *M. incognita*. Ultimately, this study will provide better insight into the temperature-dependent biology and ecological adaptation of *M. enterolobii* as well as *M. floridensis* in regions where *M. incognita* is also most likely to establish successfully.

## Materials and Methods

### Nematode culture and second-stage juvenile (J2) inocula

The nematode isolates were reared on tomato cv. BHN 589 in the greenhouse (21 ± 8°C). *M. enterolobii*, *M. floridensis*, and *M. incognita* race 3 identification were confirmed using isozyme phenotypes, DNA analysis, and host differentials, respectively (Dickson et al., 1971; Brito et al., 2008; Smith et al., 2015; Subbotin, 2019). Nematode eggs were extracted from infected roots according to established protocol (Hussey and Barker, 1973) with further modifications (Boneti and Ferraz, 1981). Egg suspension was poured through a wire mesh lined with moist filter paper inside a 140-mm × 25-mm polystyrene petri dish and maintained at room temperature. After 24 hr to 48 hr, second-stage juveniles were collected and used for the experiments.

### Preparation and maintenance of plant materials

Root penetration and development of the three *Meloidogyne* spp. were studied on tomato cv. BHN 589 seedlings. Seeds were germinated in a 38-cell seedling tray containing fine-grade vermiculite in a greenhouse (21 ± 8°C). Germinated seedlings were transplanted into 125 ml pots containing autoclaved sand (100%), fertilized weekly with 0.21% (w/v) 24N– 8P–16 K solution, Miracle-Gro (Marysville, OH). Four-to five-leaf-stage seedlings were transplanted to 251-ml polystyrene foam cups filled with autoclaved sand (100% sand). The test units were then placed in each of three growth chambers set at 30°C, 25°C, and 30°C to 25°C and maintained for 1 week before inoculation. During the experimental period, individual plants received 40 ml of water daily or as needed and fertilized biweekly as above.

## Growth chamber

Three growth chambers (Percival I-36LL; Percival Scientific, Perry, IA) were each set at 30°C, 25°C, or alternating 30°C to 25°C with a 12-hr light period at 30°C and a 12-hr dark period at 25°C. Lighting was provided by fluorescent lamps (65 μmol ⋅ m^−2^ ⋅ s^−1^). Temperature in chambers were recorded with two pendant data loggers (HOBO MX2202; Onset Computer, Bourne, MA) set to record hourly averages from 5-min sampling intervals. Hourly temperatures were averaged from the two data loggers before calculating the degree-days (DD) as described below.

### Nematode inoculation, penetration, and life cycle observations

**Figure 1:**
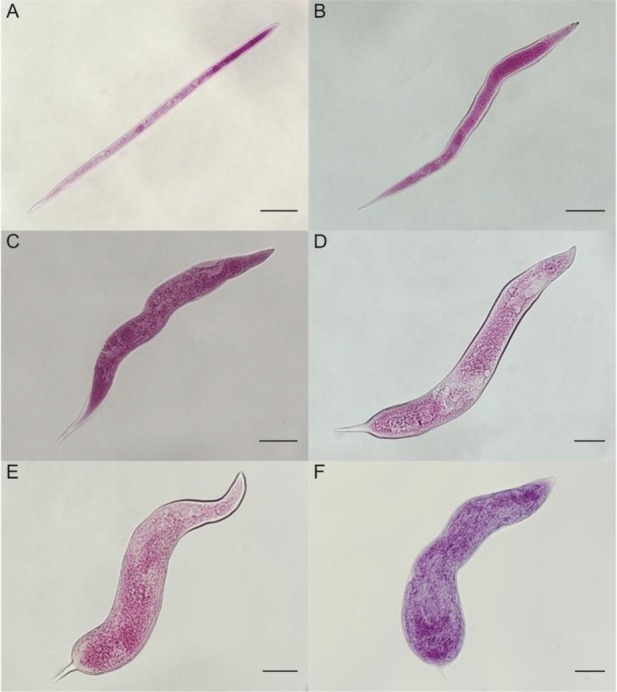
Growth stages of second-stage juveniles (J2) of *Meloidogyne* spp. (A) vermiform, early J2 with no swelling; (B, C, D) mid-stage J2 with early swelling and conoid tail; and (E, F) late J2 with swollen body, rounded terminus. Scale bars: A–E = 50 μm; and F = 10 μm.

**Figure 2:**
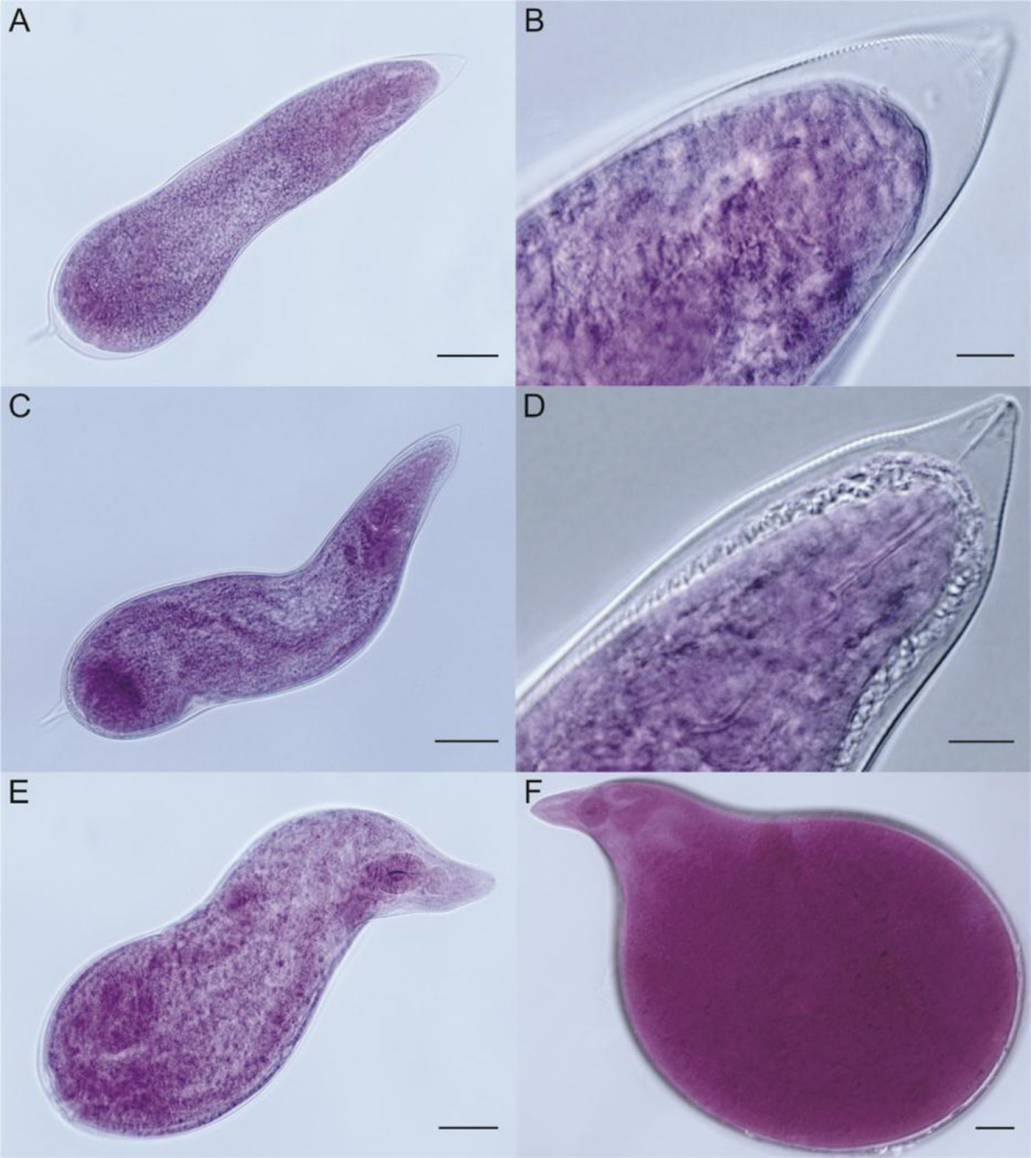
Developmental stages of *Meloidogyne* spp.; (A) third-stage juvenile (J3); (B) anterior part of a J3; (C) fourth-stage juvenile (J4); (D) anterior part of a J4; (E) female; and (F) egg-laying globose female. Scale bars: A, E, and F = 50 μm; B, C, and D = 10 μm.

Nematode inocula (200 J2/tomato seedling for *M*. *enterolobii* and *M. incognita*, and 100 J2/tomato seedling for *M*. *floridensis*) were pipetted into three 2-cm-deep holes around the seedling stem base and then holes were pinched closed. The low hatching obtained for *M. floridensis* at the time of inoculation resulted in using a different number of J2; therefore, J2 root penetration was calculated based on percentage and not numbers of nematodes that penetrated. After 48 hr from inoculation, the seedlings were removed from containers and sand around the roots was washed away under running tap water to eliminate non-penetrated juveniles. The seedlings were again transplanted into fresh autoclaved sand in 251-ml polystyrene foam cups and returned to the growth chambers. They were incubated up to 29 d in each of three growth chambers with its designated temperature treatment. Nematode development was examined at 2-d intervals between 5 d and 29 d after inoculation (DAI) with a total of 13 intervals. At each interval, two infected plants were arbitrarily collected to represent each of the three RKN species per temperature regime. A total of 78 seedlings (2 × 3 × 13) were examined for each RKN species, giving a grand total of 234 evaluated seedlings. Roots were gently rinsed and subjected to a root clearing-staining method (Byrd et al., 1983). The number of nematodes at each developmental stage (Figs. 1, 2) was observed and counted. The number of J2 that had penetrated at 5 DAI was used as a baseline for calculating the percentage of *M. enterolobii*, *M. floridensis*, and *M. incognita* J2 present in roots over the 29-d observation period. When globose females were detected for the first time, the root samples were stained (Thies et al., 2002) to aid with visualization of egg masses, which would indicate the emergence of egg-laying females. The egg masses were also checked to avoid missed counting of egg-laying attributable to egg masses that may have been dislodged during the process of clearing and staining of internal root tissues. Presence of egg masses, therefore, corresponded to the number of egg-laying females that were present before they were dislodged during the root-clearing and staining processes. The stained root systems were immersed in glycerol before different developmental stages were examined individually under the stereomicroscope (Zeiss Stereo Discovery.V8, Oberkochen, Germany).

### Classification of nematode developmental stages

The number of nematodes in each developmental stage was recorded. They were assigned as J2 and succeeding stages up to new-generation vermiform J2. The J2 present in the roots beginning at 5 DAI were classified further into three growth stages based on their body shape as follows: (i) early stage J2 − vermiform with no swelling, (ii) mid-stage J2 − with early swelling and conoid tail, and (iii) late J2 − with swollen body and rounded terminus. Third- and fourth-stage juveniles were distinguished based on the cuticle layers in the anterior part of their body as previously described (Triantaphyllou and Hirschmann, 1960; Dávila-Negrón and Dickson, 2013). To distinguish between stages J3 and J4, the nematodes were handpicked and mounted in glycerin on glass slides (25 mm × 75 mm × 1 mm) for observation at ×40 magnification individually under the stereomicroscope.

## Data collection and analyses

Observations were made on whole root systems from two tomato plants for each treatment, for a total of 18 observations at each time point between 5 d to 29 d. For each, the total number of nematodes present in whole root system was used as the baseline for calculating the percentage of nematodes at each of the following developmental stages: J2, J3, J4, females, and egg-laying females. The percentages were averaged for the two observations and the resulting values were plotted on the graph. The percentage of penetrated J2 at 5 DAI was calculated by dividing the number of J2 embedded in the roots by the number of inoculated J2. To assess the main and interaction effects of RKN species and temperature treatment on nematode infectivity, data on proportion of penetrated J2 at 5 DAI were subjected to two-way (RKN species × temperature treatment) analysis of variance (ANOVA) using SigmaPlot. Tukey’s HSD test (*P* ≤ 0.05) was used to compare means. To calculate the accumulated degree-days (ADD) for vermiform J2 to reach each successive developmental stage or to complete a generation, the daily difference between mean temperature in the growth chamber and the base temperature (T_b_) was summed over the number of DAI. The mean T_b_ of *M. incognita* was 9.8°C when inoculated on okra (Dávila-Negrón and Dickson, 2013), 10.1°C on tomato (Ploeg and Maris, 1999), and 10.1°C on clover (Vrain et al., 1978). To date, there have been no temperature-based models developed for *M. enterolobii* and *M. floridensis* to estimate the T_b_ but we suspect similarities with the heat requirements for *M. incognita* development and those of other tropical and subtropical RKN species (Ferris et al., 1978; Maleita et al., 2012); thus, we followed Tyler’s (1933) calculation of heat units for RKN development, wherein each centigrade above 10°, acting for 1 hr, is counted as one effective unit.

## Results

### Temperature effects on root penetration

**Figure 3:**
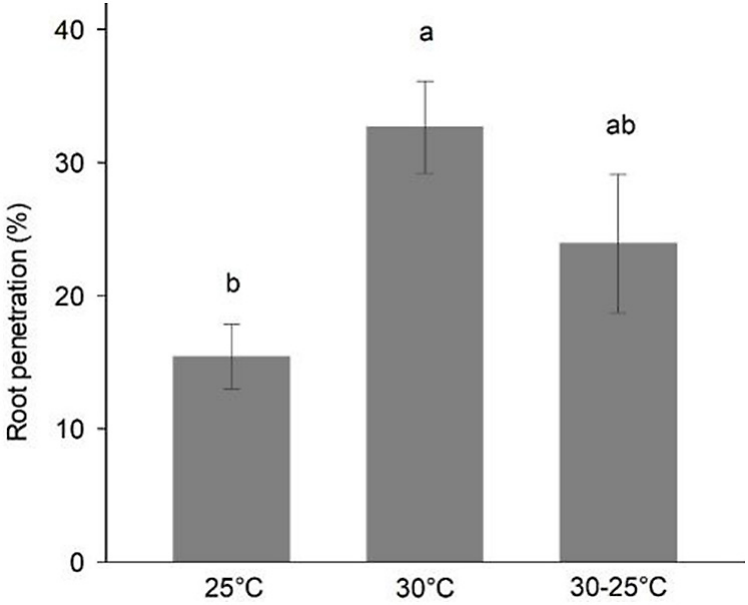
Percentage of *Meloidogyne* spp. second-stage juveniles (J2) penetrating tomato roots grown under different temperature (25°C, 30°C, or 30-25°C [12-hr light period at 30°C and 12-hr dark period at 25°C]) at 5 DAI. Bars represent the grand means ± SE of the three *Meloidogyne* spp. (*M. enterolobii, M. floridensis*, and *M. incognita* race 3). The percentage of penetrated J2 was calculated by dividing the total number of nematodes observed in the whole root system at 5 DAI over the initial inoculum concentration. Bars with different letter(s) indicate significant differences based on Tukey’s HSD test (*P* ≤ 0.05). DAI, days after inoculation.

No significant interaction was found between RKN species and temperature treatments for the number of J2 that penetrated the root system. Based on the average of the three nematode species evaluated, the percentage of J2 that penetrated the whole root system of tomato at 5 DAI ranged from 15% to 33% under the three temperature regimes. Root invasion of J2 in tomato roots was affected by temperature regardless of the species (Fig. 3). For all three species, the percentage of J2 penetrating roots at 5 DAI was greater at 30°C than at 25°C, and intermediate at 30°C to 25°C (*P* = 0.022).

### Temperature effects on post-penetration development

**Figure 4:**
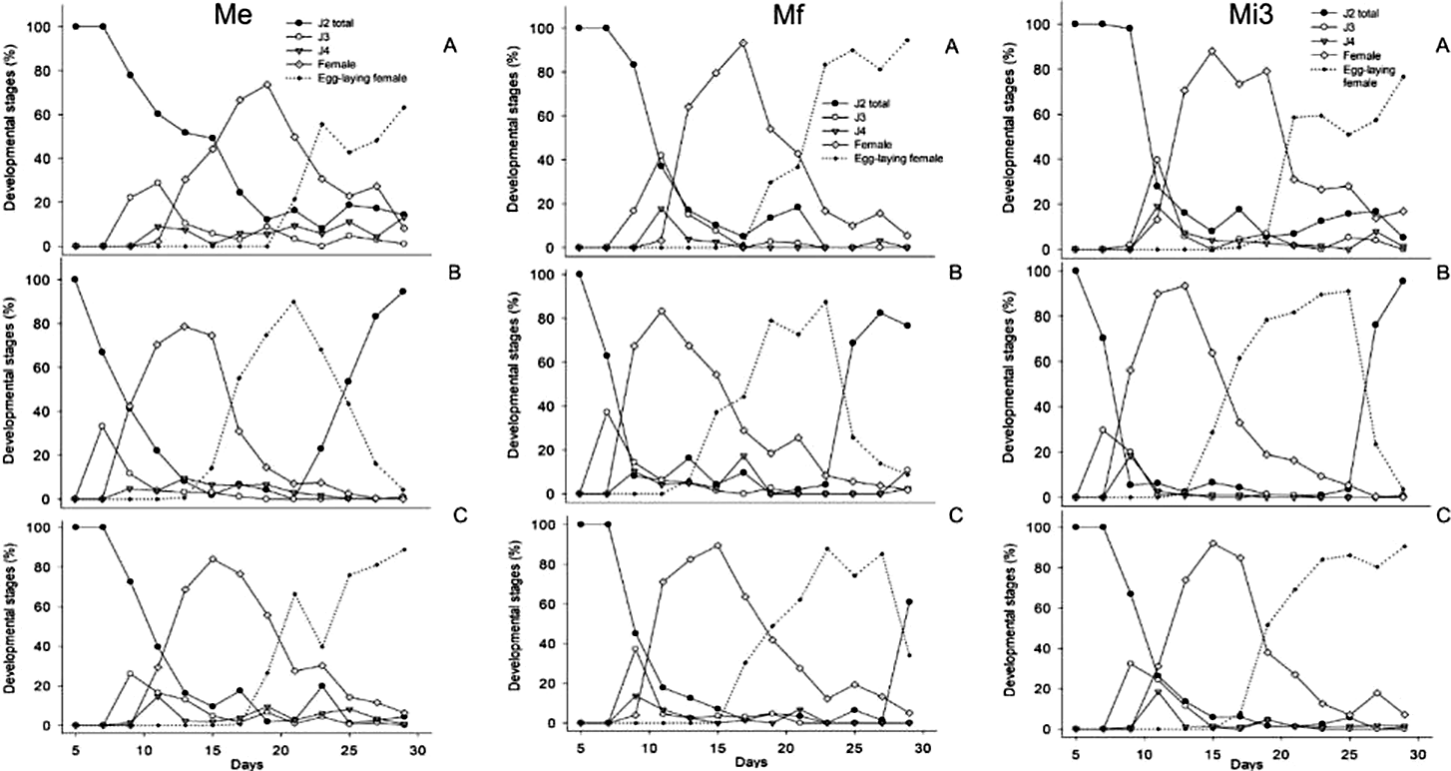
Percentage of *Meloidogyne enterolobii* (Me), *M. floridensis* (Mf), and *M. incognita* race 3 (Mi3) developmental stages on tomato grown in a growth chamber for 29 DAI at 25°C (A), 30°C (B), and 30–25°C (C) (12-hr light period at 30°C and 12-hr dark period at 25°C). Percentage of nematodes in each developmental stage was based on the total number of nematodes observed in the whole root system. A total of 78 root systems per RKN species were subjected to analysis. The increase of J2 at 30°C at 23 DAI for *M. enterolobii*, at 25 DAI for *M. floridensis* and *M. incognita*, and at 30–25°C at 29 DAI for *M. floridensis* represent the earliest observation of vermiform juveniles from the second generation. RKN, root-knot nematode; DAI, days after inoculation.

During the time span of 29 d, the developmental rates of *M. enterolobii*, *M. floridensis*, and *M. incognita* varied in response to temperatures (Fig. 4). Regardless of the species, only J2 were observed inside roots at 5 DAI and 7 DAI at 25°C (Fig. 4A) and 30°C to 25°C (Fig. 4C), respectively. At 7 DAI, however, development into J3 had begun for all three species at 30°C (Fig. 4B), 2 d ahead of those at 25°C, and 30°C to 25°C with greater J3 numbers in the latter. At 11 DAI at 25°C, development into J4 and females occurred concomitantly with the increase in the numbers of J3. At 11 DAI at 30°C to 25°C, however, there was a corresponding decrease in the numbers of J3 as they increasingly developed into J4 and females; the same occurred 2 d earlier (9 DAI) at 30°C. The percentage of females increased over time at all three temperatures (Fig. 4), but occurred faster, and the number of females was greater at 30°C (Fig. 4B). At 30°C, the number of females increased from 40% to 80% for *M. enterolobii* and 60% to 95% for *M. incognita* between 9 DAI and 13 DAI, whereas for *M. floridensis* an increase of 70% to 80% occurred 2 d earlier (9–11 DAI). At 15 DAI under the same temperature (Fig. 4B), the number of females reached more than 90% for all three species. *M. floridensis* was the first to reach female stage (9 DAI), but for all three species egg-laying females were observed at 17 DAI. Egg-laying females were first observed at 13 DAI under 30°C (Fig. 4B) and at 17 DAI under 30°C to 25°C (Fig. 4C) for all three species, with, however, greater numbers occurring for *M. floridensis*. At 25°C (Fig. 4A), egg-laying females were first seen at 21 DAI for *M. enterolobii*, and at 17 DAI for *M. floridensis* and *M. incognita*. Predominance of the egg-laying female stage was apparent 17 DAI at 30°C for all three species (Fig. 4B), 19 DAI at 30°C to 25°C for *M. floridensis* and *M. incognita* (Fig. 4C), 21 DAI at 30°C to 25°C for *M. enterolobii* (Fig. 4C), 23 DAI at 25°C for *M. enterolobii* and *M. floridensis* (Fig. 4A), and 21 DAI at 25°C for *M. incognita* (Fig. 4A).

### DD required for development and life cycle completion

Cumulative days (CD) and ADD (DD; T_b_ = 10°C) required for the first observation of each different development stage from infective J2 to new-generation vermiform J2 in tomato at 25°C, 30°C, and 30°C to 25°C are shown in Table 1. At 25°C, *M. enterolobii* required more DD (308.3) to develop into egg-laying females compared with *M. incognita* and *M. floridensis* (248.1). At 30°C, the three species reached all developmental stages faster than at other temperatures (Fig. 4), but there was no difference in DD required for development from J3 to egg-laying female among the three species (Table 1).

**Table 1:** CD and ADD required for first observation of each developmental stage of *Meloidogyne enterolobii* (Me), *M. floridensis* (Mf), and *M. incognita* race 3 (Mi3) on tomato inoculated with second-stage juveniles at different temperatures.

Meloidogyne spp.	Developmental stages^a^	Temperature (^°^C)					
		25		30		30–25^b^	
		
		CD	ADD^c^	CD	ADD	CD	ADD
*M. enterolobii*	J3	9	130.6	7	152.6	9	144.1
	J4	11	159.4	9	195.7	9	144.1
	Female	11	159.4	9	195.7	11	176.1
	Egg-laying female	21	308.3	13	285.7	17	272.7
	New-vermiform J2	na^d^	na	23	506.9	na	na
*M. floridensis*	J3	9	130.6	7	152.6	9	144.1
	J4	11	159.4	9	195.7	9	144.1
	Female	11	159.4	9	195.7	9	144.1
	Egg-laying female	17	248.1	13	285.7	17	272.7
	New-vermiform J2	na	na	25	552.3	29	468.8
*M. incognita*	J3	9	130.6	7	152.6	9	144.1
	J4	11	159.4	9	195.7	9	144.1
	Female	11	159.4	9	195.7	11	176.1
	Egg-laying female	17	248.1	13	285.7	17	272.7
	New-vermiform J2	na	na	25	552.3	na	na

a J3 = third-stage juvenile; J4 = fourth-stage juvenile; new-vermiform J2 = second-stage vermiform juvenile from the second generation. ^b^Temperature alternated between 30°C and 25°C (12-hr light period at 30°C and 12-hr dark period at 25°C). ^c^ADD above a threshold temperature [base temperature (T_b_ = 10°C)]. ^d^na = none observed within the 29-d period. DD, degree-days; ADD, accumulated degree-days; CD, cumulative days.

At 25°C with 425.4 DD (T_b_ =10°C), the three species were not able to complete their life cycle (J2–J2) within 29 d, as ascertained based on the absence of new generation of vermiform J2 inside roots (Table 1). Similar results were observed at 30°C to 25°C for *M. enterolobii* and *M. incognita*, but not for *M. floridensis*, wherein the life cycle completion (J2– J2) occurred at 29 DAI (468.8 DD). However, at 30°C, the three species completed their life cycle within 23 DAI to 25 DAI with 506.9 DD for *M. enterolobii*, and 552.3 DD for both *M. floridensis* and *M. incognita*. At this temperature, new-vermiform juveniles from the second generation could be observed in the roots after 12 d (266.6 DD_30_) for *M. floridensis* and *M. incognita* and 10 d (221.2 DD) for *M. enterolobii* from the first occurrence of egg-laying females. At 30°C to 25°C, it occurred after 12 d (196.1 DD) for *M. floridensis*, but not for the other two species.

## Discussion

The infectivity and rates of development of *M. enterolobii*, *M. floridensis*, and *M. incognita* on tomato roots were affected by temperature. Greater numbers of J2, females, and egg-laying females were observed at 30°C than at 25°C or 30°C to 25°C. The infectivity in host roots requires considerable activity by the J2, and elevated temperatures would increase their activity (Tyler, 1933) up to a certain threshold, beyond which higher temperatures would be harmful or lethal to the juveniles (Wang and McSorley, 2008).

Our results indicate that all three species accelerate their developmental rate with increasing temperature and there was no difference in their development time from J2 to egg-laying females at 30°C. At this temperature, there was a reduction in the number of days taken to reach J3, J4, females, and egg-laying females, whereas lower temperature (25°C) delayed progression of the J2 into the reproductive stages, which is consistent with previous studies (Dávila-Negrón and Dickson, 2013; Vela et al., 2014).

Egg-laying females were observed at 13 DAI at 30°C for all three species, similar to previous findings for *M. incognita* on tomato at the same temperature (Davide and Triantaphyllou, 1967), but differed by 2 d (15 DAI) in another study on okra (Dávila-Negrón and Dickson, 2013). In the present study, egg-laying females were predominant at 17 DAI at 30°C for all three species and reached 90% between 21 d and 25 d. Dávila-Negrón and Dickson (2013) reported a smaller number of egg-laying females (60%) for *M. incognita* at the same temperature by the end of their observation (31 d). However, the differences in these results may be related to the different nematode isolates and to the host used in our experiment.

The duration of the life cycle of *M. enterolobii*, *M. floridensis*, and *M. incognita* was affected by temperature, as reported for other RKNs (Zhang and Schmitt, 1995; Ploeg and Maris, 1999; Maleita et al., 2012; Khan et al., 2014; Vela et al., 2014). For *M. incognita*, the life cycle (from J2 to J2) was completed on tomato plants in 20 d and 27 d at average temperatures of 30°C and 25°C, respectively (Ploeg and Maris, 1999). In the present study, *M. incognita* was able to complete its life cycle in 25 d at 30°C, but not at 25°C within our timeframe of 29 DAI.

*Meloidogyne floridensis* also completed its life cycle in 25 d at 30°C, whereas *M. enterolobii* completed it at 23 DAI. The alternating temperature (30°C to 25°C) affected the length of the life cycle for the three species by delaying their development into egg-laying females. When exposed to fluctuating temperatures, only *M. floridensis* completed its cycle at 29 DAI when new-generation vermiform J2 were observed in roots. These findings may be attributed to differences in days required for embryogenesis and hatching (Dávila-Negrón and Dickson, 2013). To our knowledge, this is the first detailed report of the development and duration of *M. enterolobii* and *M. floridensis* life cycle completion on tomato; and, given the global distribution of *M. enterolobii* and increased distribution of *M. floridensis* in the USA, these are worth further investigation under other diurnal temperature ranges and with a broader timespan.

The base temperature (T_b_) and thermal requirements or DD have varied only slightly among studies on development and life cycle of RKNs because of adaptation of these species to similar warmer climates (Trudgill et al., 2005). The calculated values of T_b_ and thermal-time requirements for egg mass formation on tomato (T_b_ = 9.8, DD = 300; Dávila-Negrón and Dickson, 2013) and on cucumber (T_b_ = 12.2, DD = 294; Giné et al., 2014) were similar to those reported for *M. incognita*. In our study, using 10°C as base temperature for calculating the DD (Tyler, 1933; Trudgill, 1995), the results from *M. enterolobii* and *M. floridensis* (DD = 248–308) lie within close ranges as that from *M. incognita*. Similar values were also reported for the life cycle of *M. incognita* (T_b_ = 10.1, DD = 400; Ploeg and Maris, 1999), *M. javanica* and *M. hapla* (T_b_ = 12.9, DD = 350; T_b_ = 8.25, DD = 554, respectively; Trudgill and Perry, 1994), and *M. hispanica* (T_b_ = 10.22, DD = 515.46; Maleita et al., 2012) on tomato, and for *M. incognita* and *M. javanica* on cucumber (T_b_ = 11.4, DD = 500; Giné et al., 2014). Using 10°C as T_b_, the values of DD calculated for the three species (*M. enterolobii* = 506.9; *M. floridensis* and *M. incognita* = 552.3) for life cycle completion from J2 to new generation of J2 at 30°C were similar to those reported for other RKN species. These results confirm that *M*. *enterolobii* and *M*. *floridensis* can reproduce in climates optimal for *M. incognita*, reportedly the most widespread RKN species worldwide (Taylor and Sasser, 1978), and further suggest that climates closer to or at 30°C could favor a shorter generation time for *M. enterolobii*.
